# Guanylate‐binding proteins: emerging critical functions in plant development, immunity and stress response

**DOI:** 10.1111/nph.70524

**Published:** 2025-08-29

**Authors:** Lucie Fernandez‐Lochu, Martine Lemaire‐Chamley, Christian Chevalier

**Affiliations:** ^1^ INRAE, Université de Bordeaux UMR1332 Biologie du Fruit et Pathologie Villenave d'Ornon F‐33140 France

**Keywords:** biomolecular condensates, Dynamin Superfamily Protein, guanylate‐binding protein, innate immunity, large GTPase, plant development, stress

## Abstract

Guanylate‐binding proteins (GBPs) are large GTPases that belong to the Dynamin Superfamily Protein family. In humans, GBPs are well‐characterized interferon‐induced GTPases, playing a central role in cell‐autonomous innate immunity against infections, inflammation and cancer. GBPs orthologs have been identified in plants only recently. Despite the limited number of reports describing their functional characterization, plant GBPs share common structural and biological features, as well as functions, with animal GBPs. This review aimed at highlighting the recent knowledge acquired on the critical role of GBPs and the associated mechanisms in plant developmental processes and in plant immunity to biotic aggressions through the control of gene expression.

## Introduction

Guanylate‐binding proteins (GBPs) are large GTPases (LGs) that belong to the Dynamin Superfamily Proteins (DSPs; Ramachandran & Schmid, 2018). DSPs are mechano‐enzymes characterized by a conserved N‐terminal globular domain harboring the GTPase catalytic activity, followed by a highly variable extension providing various functional specializations (Jimah & Hinshaw, 2019). DSPs mediate membrane remodeling in several cellular processes through membrane fusion, fission and vesiculation (Jimah & Hinshaw, 2019), and cytoskeleton remodeling, especially in the context of cytokinesis (Konopka *et al*., 2006).

Among DSPs, the innate immune response to pathogens is another well‐characterized function, mostly assigned to GBPs. GBPs are interferon‐induced GTPases, whose founding member HsGBP1 was identified in human fibroblasts following interferon treatment (Cheng *et al*., 1983). Since this discovery, hundreds of reports have described the implication of GBPs in cell‐autonomous innate immunity conferred against viral, bacterial and parasitic infections, as well as in inflammatory response and cancer.

GBPs orthologs were identified in plants only recently. The first article reporting the functional characterization of a plant GBP in tomato was published in 2020 (Musseau *et al*., 2020). Since then, only five papers have been released, describing the function of GBPs in *Arabidopsis*. Despite this scarcity, the literature shows nevertheless that plant GBPs share common structural and biological features, as well as functions, with animal GBPs (Huang *et al*., 2019). This review aimed at highlighting the recent knowledge acquired on the critical role of GBPs in plant developmental processes, and in plant immunity to biotic aggressions through the control of gene expression.

## Structure, conformation and subcellular localization of GBPs

### Protein structure and conformation

**Fig. 1:**
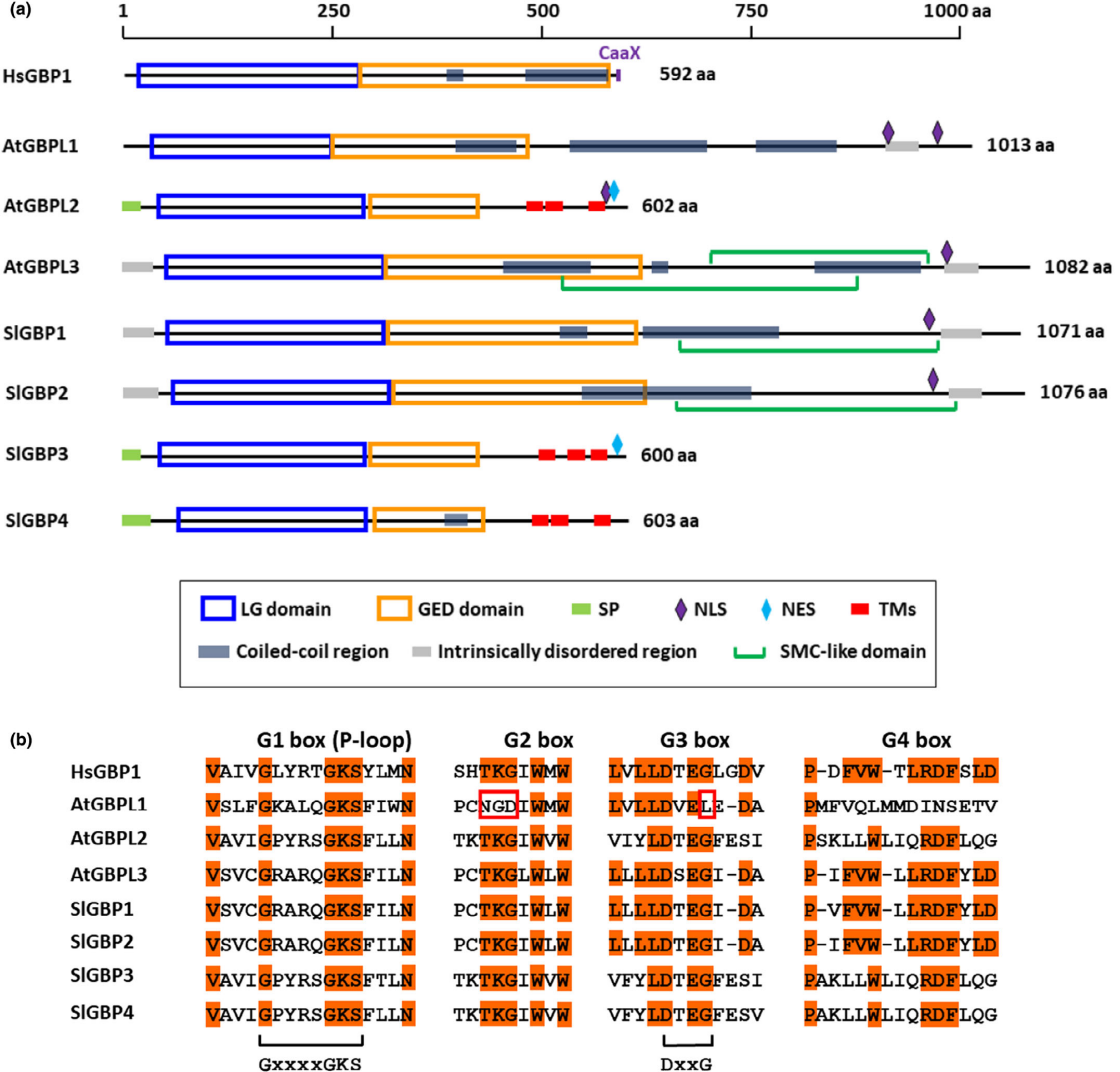
Domain structure of human HsGBP1, *Arabidopsis thaliana (At)* and tomato (*Solanum lycopersicum*, *Sl*) GBPs. (a) Domains and sequence motifs were predicted using the InterPro browser (https://www.ebi.ac.uk/interpro/protein/UniProt/). NLSs were predicted using the NLS‐Mapper tool (https://nls‐mapper.iab.keio.ac.jp/cgi‐bin/NLS_Mapper_form.cgi). SMC‐like domain were predicted using the NCBI BlastP tool. LG domain, large GTPase domain; GED domain, GTPase effector domain; CaaX, isoprenylation motif; NES, nuclear export signal; NLS, nuclear localization signal; SMC‐like domain, Structural Maintenance of Chromosome‐like domain; SP, signal peptide; TMs, transmembrane domains; aa, amino acid; GBP, guanylate‐binding proteins. (b) Alignment of GTPase (G) domains in HsGBP1, *Arabidopsis* and tomato GBPs. The G domain conferring nucleotide binding and hydrolysis is composed of four distinct structurally conserved motifs (G boxes; Jimah & Hinshaw, 2019). The G1 box or phosphate‐binding loop (so‐called P‐loop; GxxxxGKS) binds the α‐ and β‐phosphate of nucleotides. The G2 box includes a threonine that enables GTP hydrolysis, missing in AtGBPL1. The G3 box (DxxG) anchors to the terminal γ‐phosphate of nucleotide. The G4 box specifies the guanosine signature. Red outlines indicate mutated G2 and G3 box in the pseudo GTPase AtGBPL1. Accession numbers are as follows: HsGBP1, P32455; HsGBP5, Q96PP8; AtGBPL1, AT1G03830; AtGBPL2, AT2G38840; AtGBPL3, AT5G46070; SlGBP1, Solyc10g008950; SlGBP2, Solyc02g060770; SlGBP3, Solyc09g065810; and SlGBP4, Solyc06g084080.

**Fig. 2:**
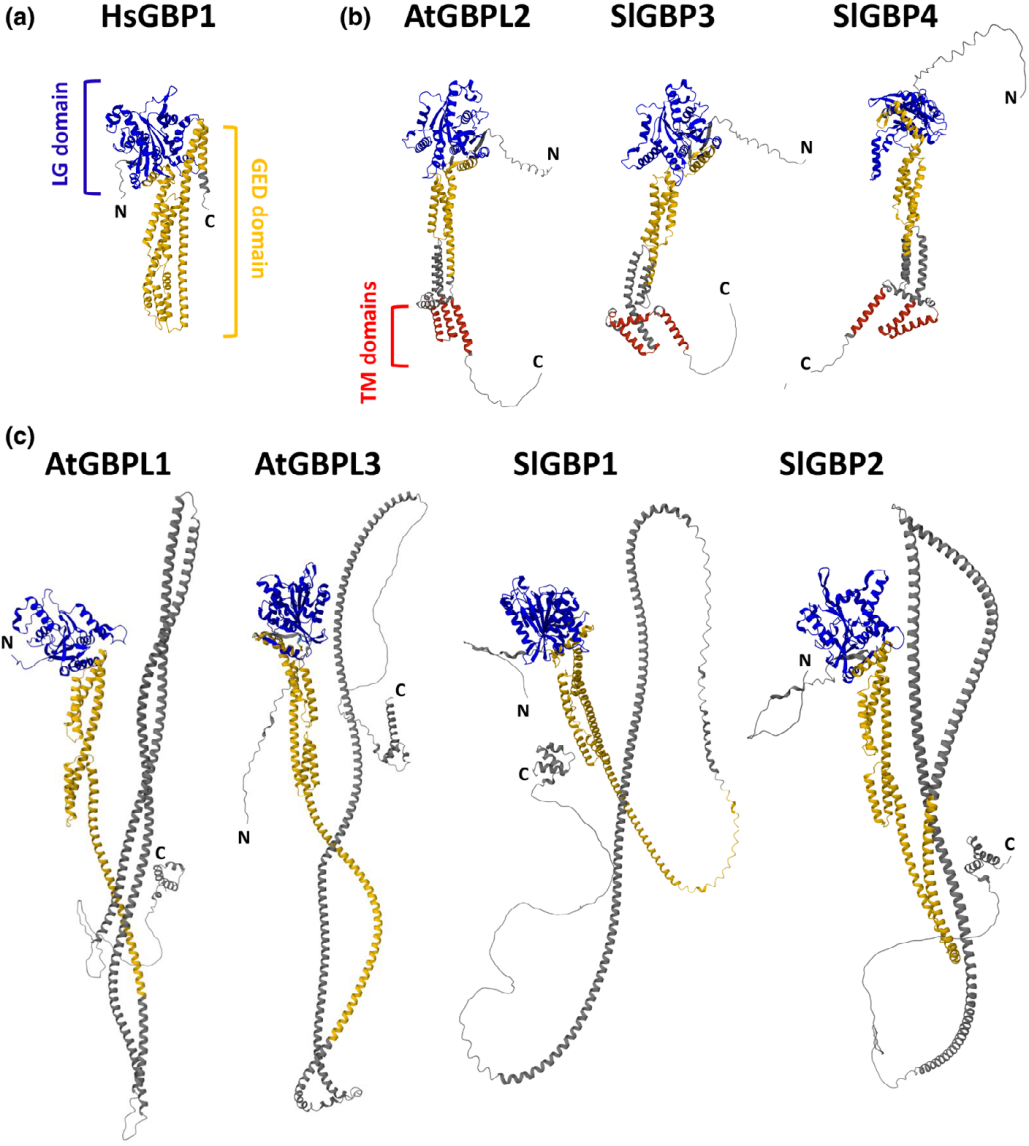
Three dimensional model prediction of the structure of (a) human HsGBP1, (b) *Arabidopsis thaliana* AtGBPL2, tomato (*Solanum lycopersicum*) SlGBP3 and SlGBP4 as representative of plant guanylate‐binding proteins (GBPs) from the AtGBPL2 clade, and (c) *Arabidopsis* AtGBPL1 and AtGBPL3, tomato SlGBP1 and SlGBP2 as representative of plant GBPs from the AtGBPL1/3 clade. Three dimensional models were predicted using the AlphaFold Protein Structure Database (https://alphafold.ebi.ac.uk/). The color code corresponds to domains illustrated in Fig. 1: blue, large GTPase (LG) domain; yellow, GTPase effector domain (GED); gray, coiled‐coil regions; red, transmembrane (TM) domains; N, N‐terminal end; C, C‐terminal end.

The human HsGBP1 has been extensively structurally and biochemically characterized, and serves as the structural model of reference for animal GBPs (Prakash *et al*., 2000). HsGBP1 is made of 592 aa (MW of *c*. 68 kDa). It is constituted of two domains: the LG N‐terminal domain and the GTPase effector domain (GED) at the C‐terminus (Fig. 1a). The LG domain is made of α‐helices and β‐sheets, and includes the catalytic sites involved in nucleotide binding and hydrolysis (Fig. 1b). In the nucleotide‐free state, the GED domain made of an elongated α‐helical structure is locked to the LG domain, resulting in a closed monomeric conformation of HsGBP1 (Prakash *et al*., 2000; Fig. 2). This state corresponds to auto‐inhibited GBP monomers with low intrinsic GTPase activity (Ghosh *et al*., 2006). GTP binding to HsGBP1 induces sequential conformational changes promoting protein dimerization via LG domains, GEDs opening and dimer mutual interaction/stabilization, leading to stimulation of GTPase activity and further GTP hydrolysis (Ghosh *et al*., 2006; Ince *et al*., 2021; Weismehl *et al*., 2024). This open conformation corresponds to an activated dimeric state of GBPs able to oligomerize to form polymers (Sistemich *et al*., 2020; Weismehl *et al*., 2024; Zhu *et al*., 2024). In the dimerized and polymerized state, some GBPs, including HsGBP1, have the unique ability to hydrolyze GTP into GMP in two consecutive hydrolysis steps (Britzen‐Laurent *et al*., 2010; Ince *et al*., 2017). Upon GTP, GDP or GMP dissociation, GBPs return to a closed monomeric conformation.

Plant GBPs display the conserved LG and GED domains (Figs 1, 2). However, structure prediction and phylogenetic analyses demonstrated that plant GBPs belong to two different clades (Reimann *et al*., 2023). A first clade, within a size range of 1000–1100 aa (110–125 kDa), is characterized by a long C‐terminal extension, with coiled‐coil regions, an intrinsically disordered region (IDR) and one or two nuclear localization signals (NLS; Huang *et al*., 2021; Reimann *et al*., 2023; Fig. 1a). This clade, termed the AtGBPL1/3 clade, encompasses the *Arabidopsis thaliana* AtGBPL1 and AtGBPL3, as well as the tomato (*Solanum lycopersicum*) SlGBP1 and SlGBP2. Within this clade, AtGBPL3 and SlGBP1/2 harbor STRUCTURAL MAINTENANCE OF CHROMOSOMES (SMC)‐like domains, found in SMC proteins (Uhlmann, 2016). SMC proteins are involved in the compaction of genomes, as well as sister chromatid cohesion and segregation during cell division (Uhlmann, 2016). Hence, the presence of SMC domains may confer to AtGBPL3 and SlGBP1/2 a function during mitosis together with the ability to bind chromatin DNA. Members of the second clade, termed the AtGBPL2 clade, encompassing AtGBPL2 and the tomato SlGBP3 and SlGBP4, resemble HsGBP1, with a size of *c*. 600 aa and a MW of *c*. 69 kDa. They possess a shorter C‐terminal extension devoid of coiled‐coil and IDR regions. This C‐terminal extension is characterized by the presence of several transmembrane (TM) domains, similarly to the animal ATLASTIN (ATL). ATL proteins are DSPs, involved in the fusion of endoplasmic reticulum (ER) membranes and ER–Golgi vesicle trafficking (Jimah & Hinshaw, 2019). A recent study indicated that AtGBPL2 might be the plant counterpart of animal ATL1 (Pan *et al*., 2025).

Reimann *et al*. (2023) mined more than 200 plant GBPs in a phylogenetic analysis. The number of GBP members in both clades varies according to the plant species. Additionally, the comparison of primary sequences provides a first glance at different functional roles among plant GBPs. GBPs within the AtGBPL2 clade share a high degree of amino acid identity (*c*. 70–80% overall identity). Inside of the AtGBPL1/3 clade, GBP orthologs display a 60–70% overall amino acid identity. However, the LG domain of AtGBPL1 shares only *c*. 35% identical residues with AtGBPL3, SlGBP1/2, or animal GBPs. In particular, the AtGBPL1 protein sequence within the G2‐, G3‐ and G4 box of the GTPase (G) domain is highly divergent when compared to other plant GBPs and HsGBP1 sequences (Fig. 1b). Such a mutated catalytic domain provides defective GTPase activity to AtGBPL1 (Huang *et al*., 2021). Interestingly, this characteristic of a pseudo GTPase found in AtGBPL1 seems to be exclusive to Brassicaceae species. Both AtGBPL3 and AtGBPL2 harbor GTPase activity and are able to produce GDP, but not GMP (Huang *et al*., 2021; Reimann *et al*., 2023).

Whether the biochemical and enzymatic attributes found in animal GBPs also contribute to plant GBP conformation and dimerization remains largely uncharacterized. However, AtGBPL3 is able to dimerize via the nucleotide‐dependent self‐association of the LG domain, required for GTPase activity, and via the self‐association of the GED domain (Reimann *et al*., 2023). Whether homo‐ or heterodimerization occurs *in planta* remains to be demonstrated. Yet, evidence for a direct interaction between AtGBPL3 and AtGBPL1 inside of the nucleus, and AtGBPL3 and AtGBPL2 were provided (Huang *et al*., 2021; Pan *et al*., 2025).

### Subcellular localization of GBPs

HsGBPs differ in their cellular localization according to the presence of a CaaX isoprenylation motif at the C‐terminal end serving for membrane anchoring (Fig. 1a). HsGBP1/2/5 harbor this motif and are located at membranes of various cellular compartments (Britzen‐Laurent *et al*., 2010; Sistemich *et al*., 2021). In the absence of this motif, HsGBPs are homogeneously located in the cytosol and/or the nucleus (Tripal *et al*., 2007; Britzen‐Laurent *et al*., 2010).

The binding of HsGBPs to membranes not only requires the presence of an isoprenyl moiety, but most importantly its accessibility. Indeed, under the closed monomeric conformation, the isoprenyl tail of HsGBP1 is positioned in a hydrophobic pocket of the GED domain, thus preventing its interaction with membranes (Sistemich *et al*., 2021). GTP binding drives the open conformation, which releases the isoprenyl tail and renders it accessible for membrane anchoring (Shydlovskyi *et al*., 2017; Sistemich *et al*., 2021). Remarkably, the HsGBP1 C‐terminal isoprenylation is required for the formation of highly polymerized structures, encompassing as much as thousands of molecules (Sistemich *et al*., 2020; Weismehl *et al*., 2024). Recently, Zhu *et al*. (2024) demonstrated the major role of these HsGBP1 conformational changes in the establishment of a host defense platform for cell‐autonomous immunity, assembling nearly 30 000 HsGBP1 molecules. Besides its ability to target membranes, it is noteworthy that HsGBP1 appears also as granular/vesicular structures distributed throughout the cytoplasm, which are associated exclusively with the cytoskeleton (Ostler *et al*., 2014; Tipton *et al*., 2016).

**Fig. 3:**
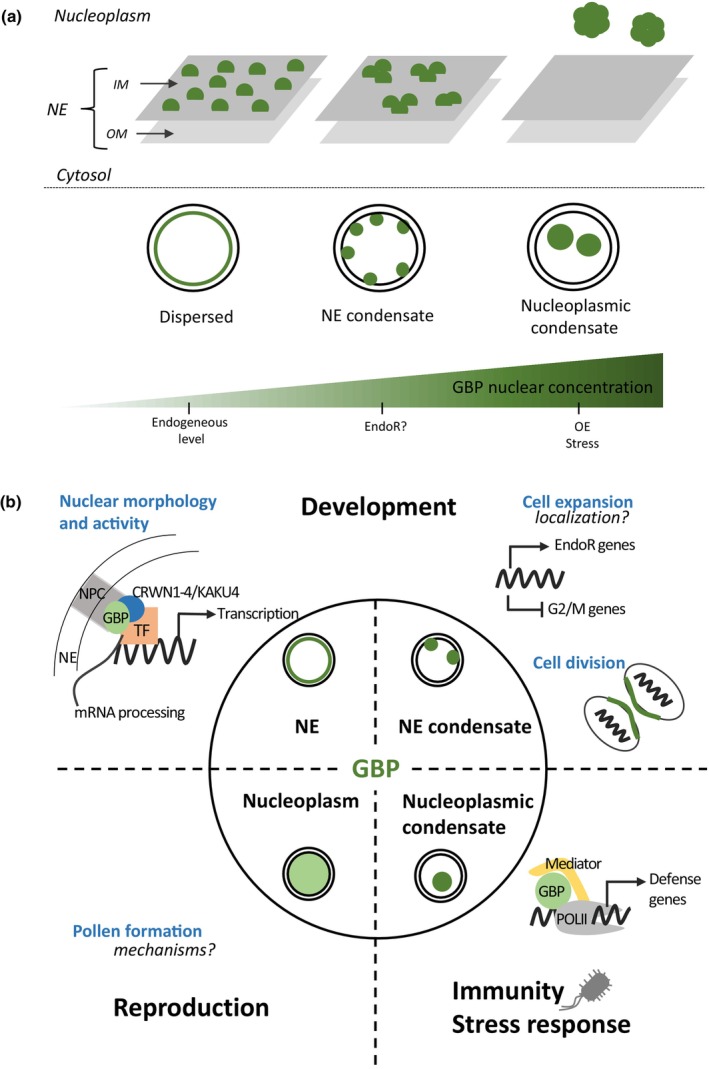
Subnuclear localization and multifunctional roles of plant guanylate‐binding proteins (GBPs) of the AtGBPL1/3 clade. (a) The ability of plant GBPs to drive liquid–liquid phase separation (LLPS) according to their nuclear concentration redefines their subnuclear localization. At endogenous levels, GBPs are in dispersed form, mainly localized at the inner membrane of the nuclear envelope (NE). At high protein concentration, upon overexpression, stress or likely endoreduplication, GBPs form condensates of different sizes either localized at the NE (intermediate phase) or in the nucleoplasm (separated phase). (b) The subnuclear localization of GBPs is associated with diverse biological functions in plants. During development, GBPs are localized at the NE. There, GBPs participate in nuclear morphogenesis during interphase, within a complex encompassing CRWN1&4 and KAKU4, at the nuclear pore complex of the NE. The AtGBPL3/CRWN1&4/KAKU4 complex interacts with chromatin, where it recruits chromatin remodelers and participates in the regulation of gene expression and mRNA processing. GBPs are involved in the reformation of the NE during mitosis, as a result of the AtGBPL3/CRWN1&4/KAKU4 complex disassembly and the massive accumulation of AtGBPL3 into speckles at sites of active NE reformation. GBPs play a crucial role in tissue differentiation and cell fate during fruit growth by maintaining endopolyploid cells in a nonproliferative state that affects the cytoskeleton network and/or controls the expression of endoreduplication‐associated and mitosis‐associated genes, respectively, positively and negatively. During innate immunity and in response to stress, GBPs form LLPS‐driven condensates, which localize within the nucleoplasm. Within condensates, GBPs bind directly to promoters of defense‐related genes and recruit transcriptional coactivators of the Mediator complex and RNA polymerase II machinery to ensure the transcriptional reprogramming induced by immune activation. During gametophyte development and pollen grain formation, GBPs localize to the entire nucleoplasm, but the mechanisms and GBP interacting protein network are still undeciphered. Arrows and blunt‐ended arrows represent positive and negative regulation, respectively. GBPs are represented in green; NE, nuclear envelope; OM, outer membrane; IM, inner membrane; EndoR, endoreduplication; OE, overexpression; NPC, nuclear pore complex; TF, transcription factors; PolII, RNA polymerase II.

Plant GBPs lack the C‐terminal CaaX motif found in HsGBP1/2/5. However, *Arabidopsis* and tomato orthologs of the AtGBPL2 clade have an N‐terminal signal peptide and three TM helices in the C‐terminal extension (Fig. 1a), predicting their targeting and integration into the endomembrane system. Indeed, AtGBPL2 localizes to the tonoplast (Reimann *et al*., 2023), as well as to the ER (Pan *et al*., 2025), as expected for an ATL‐like protein. Although AtGBPL2 possesses an NLS in its C‐terminus, involved in nuclear import, it also presents in tandem a nuclear export signal (NES) domain responsible for its cytosolic localization, similarly to ATL1. When this NES domain is mutated, the ER localization of AtGBPL2 is abolished, and AtGBPL2 relocalizes to the nuclear envelope (NE; Pan *et al*., 2025). *Arabidopsis* and tomato homologs of the AtGBPL1/3 clade also possess one or two NLSs in their C‐terminus, but no NES domains (Fig. 1a). AtGBPL1/3 and SlGBP1/2 are indeed nuclear localized (Huang *et al*., 2021), according to different sublocalizations within the nucleus (Fig. 3a). More precisely, AtGBPL3 accumulates mainly at endogenous levels at the inner membrane of the NE, where it is enriched at the nuclear pore complex (NPC) during interphase (Tang *et al*., 2022; Reimann *et al*., 2023). During mitosis, AtGBPL3 localizes as speckles surrounding the condensing chromosomes; it then localizes to the inner hemisphere of the daughter nuclei during telophase and to the newly formed envelope during cytokinesis (Reimann *et al*., 2023). Such AtGBPL3 speckles are promoted by enhanced nucleotide‐dependent self‐association of the LG domain resulting from locally stimulated AtGBPL3 GTPase activity.

Notably, the distribution of AtGBPL3 in pollen grains and pollen tubes is very singular, as it is absent from NE but accumulates homogeneously in the nucleoplasm (Reimann *et al*., 2023).

A remarkable feature of plant GBPs is their ability to form biomolecular condensates, which assemble in dynamic membrane‐less organelles through liquid–liquid phase separation (LLPS) within the nucleus (Huang *et al*., 2021). The LLPS process is driven by the association of multiple IDR‐containing proteins involved in developmental or environmental cues, such as in plant immunity and stress response (Wang & Gu, 2022; Solis‐Miranda *et al*., 2023). Even in the absence of any exposure to stress, the overexpression of *Arabidopsis* and tomato GBPs also leads to the formation of such droplet‐like condensates of variable range of size, at the nuclear periphery and/or within the nucleoplasm (Huang *et al*., 2021; Tang *et al*., 2022; Reimann *et al*., 2023; Fig. 3a). Indeed, LLPS is a function of molecule concentration, and increased GBP nuclear concentration is sufficient to induce GBP condensates (Huang *et al*., 2021). Although IDRs are necessary for the formation of GBP condensates, GTPase activity is also required. Whether LLPS‐associated GBP condensates formed upon stress or overexpression and speckles found during mitosis are similar structures remains to be determined.

Plant GBPs belonging to the AtGBPL2 and AtGBPL1/3 clades are either cytosolic or nuclear, respectively. Moreover, different subnuclear localizations have been evidenced for the AtGBPL1/3 clade that seem to influence their diverse biological functions in plants (Fig. 3b), as described in the following section.

## Multifunctional roles of GBP in plants

According to their pivotal role in cell‐autonomous innate immunity, in response to cancer, inflammation and infection (for reviews see Ngo & Man, 2017; Honkala *et al*., 2020; Kirkby *et al*., 2023), animal GBPs are described as the ‘guardians of host defense in health and disease’ (Tretina *et al*., 2019). Part of these functional roles and associated cellular mechanisms is conserved in plants, especially regarding intranuclear signaling for immunity against pathogens (Huang *et al*., 2019). Moreover, GBPs acquired specific functions in plants related to nuclear architecture and their ability to bind DNA, thus exerting a direct control on gene expression (Fig. 3b).

### Plant GBP and nuclear morphogenesis and activity

The NE localization of AtGBPL3 at endogeneous level is due to its interaction with the plant lamins CRWN1, CRWN4 and KAKU4 and the nucleoporin NUP136 of the nuclear pore basket (NB; Reimann *et al*., 2023; Tang *et al*., 2022; Fig. 3b). The nuclear lamina (also named nucleoskeleton) is a dense fibrillary network beneath the inner nuclear membrane that associates with NPC. The lamina is essential to ensure the structural integrity of the NE and specific perinuclear chromatin anchoring, thereby regulating the overall nuclear morphology and activity (Wang *et al*., 2022). AtGBPL3 recruited at the nuclear lamina in an AtGBPL3/CRWN1&4/KAKU4 protein complex is crucial for proper nuclear morphogenesis in differentiating cells during interphase. Indeed, the disruption of AtGBPL3 induces altered nuclear size and shape (Reimann *et al*., 2023), similarly to defective lamin mutants (Cao *et al*., 2024; Hu *et al*., 2019).

AtGBPL3 is not only involved in nuclear architecture, but it also plays a major part in chromatin modification and organization, transcription initiation and regulation, and RNA splicing and processing (Tang *et al*., 2022). These functions are in accordance with the role of lamina and NPC in coordinating the functional organization of the genome by anchoring a large fraction of the silent heterochromatin to the nuclear periphery (Strambio‐De‐Castillo *et al*., 2010). AtGBPL3 binds to chromatin at interphase, most likely via its SMC‐like domains (Fig. 1a), and especially at the pericentromeric heterochromatin and/or perinuclear heterochromatin, which are highly subjected to the repressive H3K27m3 histone modification (Reimann *et al*., 2023). This mechanism involves the interaction of AtGBPL3 with CRWN1 at the lamina, and PWWP DOMAIN INTERACTOR OF POLYCOMB 1 (PWO1), a Polycomb Repressive Complex 2 (PRC2) associated protein mediating the deposition of the H3K27m3 repressive mark (Tang *et al*., 2022; Reimann *et al*., 2023). However, AtGBPL3 also binds euchromatin and promoter regions of transcriptionally active genes, as it recruits both activators and repressors of gene expression (Huang *et al*., 2021; Tang *et al*., 2022). Hence, AtGBPL3 may contribute in diverse combinations with lamins to a more dynamic regulation of gene expression at NPC than only repression of heterochromatin (Fig. 3b). Plants disrupted for AtGBPL3 present stunted growth; this phenotype is enhanced in the presence of mutated lamins and NB proteins, leading to early senescence. Within this lamina/NPC network, plant GBPs emerge as novel major architectural and regulatory components of the nucleus, essential for plant vegetative growth and survival by maintaining nutrient homeostasis and suppression of stress responses under normal conditions. It is noteworthy that this function of AtGBPL3 in nuclear architecture, NE localization and transcriptional regulation at NE does not require the GTPase activity (Reimann *et al*., 2023).

In the next section, additional roles of nuclear GBPs during plant development will be described in relation to their subnuclear localizations.

### GBPs and the control of cell division during plant development

AtGBPL3 is highly expressed in the root apical meristem (RAM), where it is required for proper mitosis and is thus essential for root growth (Reimann *et al*., 2023). NE breakdown at mitosis triggers the disassembly of the AtGBPL3/CRWN1&4/KAKU4 complex. However, AtGBPL3 remains extensively co‐localized with CRWN4, in the form of nuclear speckles surrounding the condensing prophase chromosomes and at the inner hemisphere of the daughter nuclei at telophase. During mitosis, AtGBPL3 is required for the reformation of the NE in the daughter cells (Reimann *et al*., 2023), likely resulting from the massive AtGBPL3 speckle accumulation at sites of active NE expansion (Fig. 3b). Although the exact role of AtGBPL3 speckles during mitosis remains unclear, Reimann *et al*. (2023) suggested a function in the recruitment of NE‐associated proteins to remodel the NE during root development. When AtGBPL3 is mutated, the RAM displays a defective tissue patterning, with fewer and severely deformed cells, as well as aberrant cell wall positioning (Reimann *et al*., 2023). AtGBPL3 mutants also exhibit altered growth in shoot (Tang *et al*., 2022), but whether these alterations result from similar mechanisms is not known. Whatever these mechanisms are, the GTPase activity and the disordered CC region seem to be essential for AtGBPL3 function in both root and shoot development (Tang *et al*., 2022; Reimann *et al*., 2023).

In tomato, SlGBP1 controls fruit growth (Musseau *et al*., 2020). Tomato fruit growth proceeds from a first intense period of cell divisions, followed by endoreduplication‐driven cell expansion, accounting for final fruit size (Mauxion *et al*., 2021; Tourdot *et al*., 2023). The disruption of SlGBP1 induces lower endoreduplication levels, thereby reducing cell sizes, and untimely abnormal cell wall patterning within the fruit tissue, resulting from the division of endoreduplicated cells (Musseau *et al*., 2020). In addition, the expression patterns of known endocycle and mitotic cycle regulators are modified (Fig. 3b). Therefore, SlGBP1 plays a crucial role in maintaining differentiated (endopolyploid) cells in a nonproliferative state, thus acting as inhibitors of cell division during fruit growth. How this repression on cell division occurs is still undeciphered. Such an antiproliferative function could involve remodeling of the cytoskeleton network, as do human GBPs during cancer (Ostler *et al*., 2014; Wandel *et al*., 2017). However, since a GBP inhibition of cell proliferation genes occurs in tomato fruit, a deactivation of the Hippo signaling pathway (Wu & Guan, 2021) could occur in plant cells, as demonstrated for the human GBPs in cancer cells (Unterer *et al*., 2018). Homologs of the TEA‐Domain transcription factor (TF), whose direct interaction with HsGBP1 leads to the deactivation of the Hippo pathway (Unterer *et al*., 2018), have not yet been identified in plants. However, plant cells do possess homologs of the upstream regulatory Hippo kinases (Musseau *et al*., 2020).

Although poorly investigated, another role of plant GBPs seems to emerge during pollen development. While *Arabidopsis* knockout mutants of AtGBPL1 and AtGBPL2 are fertile, the disruption of AtGBPL3 via CRISPR‐Cas9 gene editing or homozygous T‐DNA insertions in the coding region leads to sterility (Huang *et al*., 2021; Tang *et al*., 2022; Reimann *et al*., 2023). As AtGBPL3 is highly expressed in the male gametophyte (Reimann *et al*., 2023), this sterility is due to defective pollen grain formation, indicating that AtGBPL3 is essential for gametophyte development. Additional mechanisms associated with AtGBPL3 and the interacting protein network are likely to be involved in relation to its unique nucleoplasmic localization in pollen grain when compared to other tissues/organs (Reimann *et al*., 2023; Fig. 3b).

Plant GBPs thus display multifaceted roles during cell division either as a positive actor, likely establishing a structural basis for molecule tethering, or as a negative regulator via gene expression control, both required for proper tissue patterning. In the following section, we will address how GBPs turn out to be major players in biotic and abiotic stress responses.

### GBPs in plant immunity and response to stress

Originally, AtGBPL1 and AtGBPL3 were identified from a proteomic analysis aimed at isolating IDR‐containing proteins that could form LLPS‐driven condensates within the nucleus in response to biotic stress (Huang *et al*., 2021). Indeed, the AtGBPL3 condensates induced under bacterial infection or under overexpression confer an increased resistance to infection, compared with the *atgbpl3* hypomorphic mutants more susceptible to pathogens (Huang *et al*., 2021). Interestingly, an *atgbpl1* mutant also shows an increased resistance, which can be abolished by the introduction of the *atgbpl3* mutation. This led to the conclusion that the pseudo GTPase AtGBPL1 could be a negative allosteric regulator of AtGBPL3. The immune activation triggers an allosteric switch where AtGBPL1 no longer sequesters the catalytically active AtGBPL3 (Huang *et al*., 2021). As a result, AtGBPL3 is free to reallocate to the nucleoplasm where it concentrates in biomolecular condensates, called GBPL defense‐activated condensates (GDACs; Huang *et al*., 2021; Fig. 3a). AtGBPL3 in condensate form binds directly to promoters of defense‐related genes and recruits transcriptional coactivators of the Mediator complex and RNA polymerase II machinery to ensure the transcriptional reprogramming induced by immune activation (Fig. 3b). Among these defense‐related genes, AtGBPL3 is recruited to the promoters of genes involved in the salicylic acid (SA) pathway, a major hormone mediating plant defense against pathogens. These genes include *ICS1*, which encodes ISOCHORISMATE SYNTHASE 1, the key enzyme for SA production (Huang *et al*., 2021). In addition, AtGBPL3 within GDACs regulates the transcription of two functionally redundant TFs that bind to the promoter of *ICS1* in response to SA, namely CALMODULIN‐BINDING PROTEIN 60 g (CBP60g) and SYSTEMIC ACQUIRED RESISTANCE DEFICIENT 1 (SARD1; Kim *et al*., 2022).

The formation of nuclear GDACs is reduced at elevated growth temperature, which suppresses the recruitment of AtGBPL3 to the *CBP60g* and *SARD1* promoters, impairing SA biosynthesis (Kim *et al*., 2022). This discovery shed light on the temperature vulnerability of the SA pathway (Castroverde & Dina, 2021), and the impact of heat stress on basal and pathogen‐induced immunity (Huot *et al*., 2017). Some GBP condensates are temperature‐insensitive and target different gene promoters, but their role in stress response is not yet established (Kim *et al*., 2022). As smaller droplets fuse into larger droplets during LLPS according to molecule concentration (Huang *et al*., 2021; Fig. 3a), it is likely that different GBP condensate types coexist according to nuclear protein concentration and interacting proteins, with possibly different roles during plant development.

A role in immunity was recently evidenced for plant cytosolic GBP of the AtGBPL2 clade (Pan *et al*., 2025). AtGBPL3, with AtGBPL2, belongs to a signaling pathway that acts downstream of *CONSTITUTIVE EXPRESSION OF PR GENES 5* (*CPR5*) to modulate plant immunity and counteract the NPC‐located nucleoporin CPR5 function. CPR5 acts as a key negative regulator of plant effector‐triggered immunity (ETI; Wang *et al*., 2014) in preventing nuclear import of immune signals and core cell‐cycle inhibitors, thereby inactivating the immune response and induced programmed cell death (PCD; Zhang *et al*., 2025). Plants with disrupted CPR5 function exhibit elevated SA content, auto‐immune activation and spontaneous PCD, resembling ETI. A gain‐of‐function mutation that disrupts the NES motif present in AtGBPL2 (Fig. 1a) was identified as a suppressor of the *cpr5* mutation. As a consequence, the mutated AtGBPL2 is reallocated from the ER to the NE, where it activates AtGBPL3 via its GTPase activity, thereby suppressing the *cpr5* mutant phenotype (Pan *et al*., 2025).

Although the function of the wild‐type AtGBPL2 in immunity is still undeciphered, these findings stress how important the subcellular compartimentalization of plant GBPs is in regulating the plant immune response. Indeed, NES‐containing GBPs from the AtGBPL2 clade are prone to being exported from the nucleus despite the presence of an NLS motif. In the absence of a functional NES, these GBPs are susceptible to intervening in biological processes close to that assigned to GBPs from the AtGBPL1/3 clade, *that is* development and plant immune response, thus modifying their native function. Therefore, plant cytosolic (ER) GBPs are likely to share the same processes with nuclear‐located GBPs, most probably via different mechanisms, but according to intertwined pathways, as to optimize a trade‐off between plant growth and immunity.

## Conclusions and future directions

Despite evolutionary acquired differences in protein structure and conformation, as well as in subcellular localization, animal and plant GBPs do conserve functional similarities resulting from adaptations within the two phyla. The GTPase activity, the dimerization and formation of highly polymeric structures, the interaction with multiple protein partners and formation of multivalent protein complexes are among the conserved biochemical properties. These properties provide GBPs multifunctional roles in response to developmental and stress stimuli, and in cell‐autonomous innate immunity. GBPs participate in the regulation of cell division with pro‐ and antiproliferative activity, in cell differentiation and organismal development, leading to diseases in animals and altered tissue patterning in plants if impeded. Plant GBPs are able to mediate membrane‐ and/or cytoskeleton remodeling, an activity typical of DSP LGs.

However, the ability of AtGBPL3 to interact with chromatin, where it recruits chromatin remodelers, and participates in the regulation of gene expression and mRNA processing, represents the greatest difference between plant and animal GBPs reported so far. In plant immune response, GBP condensates represent a cellular centralized organization aimed at regulating gene expression to protect against phytopathogen infection. This function in plant immunity involves the regulation of SA signaling, a major hormone in plant responses to biotic and abiotic stress, and in plant physiology and development (Rivas‐San Vicente & Plasencia, 2011; Spoel & Dong, 2024). Whether GBP condensates may assemble in the context of abiotic stresses and regulate other cellular processes in plant growth and development will be worth investigating.

So far, the understanding of the biological functions of plant GBPs is only at its dawn. New roles and mechanisms will arise from further investigations addressing, for instance, pollen grain formation or the still poorly characterized GBPs from the AtGBPL2 clade.

## Competing interests

None declared.

## Author contributions

LF‐L and CC conceptualized and designed the manuscript. LF‐L, ML‐C and CC wrote the manuscript.

## Disclaimer

The New Phytologist Foundation remains neutral with regard to jurisdictional claims in maps and in any institutional affiliations.

## References

[nph70524-bib-0001] Britzen‐Laurent N , Bauer M , Berton V , Fischer N , Syguda A , Reipschläger S , Naschberger E , Herrmann C , Stürzl M . 2010. Intracellular trafficking of guanylate‐binding proteins is regulated by heterodimerization in a hierarchical manner. PLoS ONE 5: e14246. 21151871 10.1371/journal.pone.0014246 PMC2998424

[nph70524-bib-0002] Cao Y , Yan H , Sheng M , Liu Y , Yu X , Li Z , Xu W , Su Z . 2024. Nuclear lamina component KAKU4 regulates chromatin states and transcriptional regulation in the Arabidopsis genome. BMC Biology 22: 80. 38609974 10.1186/s12915-024-01882-5 PMC11015597

[nph70524-bib-0003] Castroverde CDM , Dina D . 2021. Temperature regulation of plant hormone signaling during stress and development. Journal of Experimental Botany 72: 7436–7458. 10.1093/jxb/erab257 34081133

[nph70524-bib-0004] Cheng YS , Colonno RJ , Yin FH . 1983. Interferon induction of fibroblast proteins with guanylate binding activity. Journal of Biological Chemistry 258: 7746–7750. 6305951

[nph70524-bib-0005] Ghosh A , Praefcke GJ , Renault L , Wittinghofer A , Herrmann C . 2006. How guanylate‐binding proteins achieve assembly‐stimulated processive cleavage of GTP to GMP. Nature 440: 101–104. 16511497 10.1038/nature04510

[nph70524-bib-0006] Honkala AT , Tailor D , Malhotra SV . 2020. Guanylate‐Binding Protein 1: an emerging target in inflammation and cancer. Frontiers in Immunology 10: 3139. 32117203 10.3389/fimmu.2019.03139 PMC7025589

[nph70524-bib-0007] Hu B , Wang N , Bi X , Karaaslan ES , Weber AL , Zhu W , Berendzen KW , Liu C . 2019. Plant lamin‐like proteins mediate chromatin tethering at the nuclear periphery. Genome Biology 20: 87. 31039799 10.1186/s13059-019-1694-3 PMC6492433

[nph70524-bib-0008] Huang S , Meng Q , Maminska A , MacMicking JD . 2019. Cell‐autonomous immunity by IFN‐induced GBPs in animals and plants. Current Opinion in Immunology 60: 71–80. 31176142 10.1016/j.coi.2019.04.017 PMC6800610

[nph70524-bib-0009] Huang S , Zhu S , Kumar P , MacMicking JD . 2021. A phase‐separated nuclear GBPL circuit controls immunity in plants. Nature 594: 424–429. 34040255 10.1038/s41586-021-03572-6 PMC8478157

[nph70524-bib-0010] Huot B , Castroverde CDM , Velásquez AC , Hubbard E , Pulman JA , Yao J , Childs KL , Tsuda K , Montgomery BL , He SY . 2017. Dual impact of elevated temperature on plant defence and bacterial virulence in Arabidopsis. Nature Communications 8: 1808. 10.1038/s41467-017-01674-2 PMC5704021 29180698

[nph70524-bib-0011] Ince S , Kutsch M , Shydlovskyi S , Herrmann C . 2017. The human guanylate‐binding proteins hGBP‐1 and hGBP‐5 cycle between monomers and dimers only. FEBS Journal 284: 2284–2301. 28580591 10.1111/febs.14126

[nph70524-bib-0012] Ince S , Zhang P , Kutsch M , Krenczyk O , Shydlovskyi S , Herrmann C . 2021. Catalytic activity of human guanylate‐binding protein 1 coupled to the release of structural restraints imposed by the C‐terminal domain. FEBS Journal 288: 582–599. 32352209 10.1111/febs.15348

[nph70524-bib-0013] Jimah JR , Hinshaw JE . 2019. Structural insights into the mechanism of dynamin superfamily proteins. Trends in Cell Biology 29: 257–273. 30527453 10.1016/j.tcb.2018.11.003 PMC9623552

[nph70524-bib-0014] Kim JH , Castroverde CDM , Huang S , Li C , Hilleary R , Seroka A , Sohrabi R , Medina‐Yerena D , Huot B , Wang J *et al*. 2022. Increasing the resilience of plant immunity to a warming climate. Nature 607: 339–344. 35768511 10.1038/s41586-022-04902-y PMC9279160

[nph70524-bib-0015] Kirkby M , Tuipulotu DE , Feng S , Lo Pilato J , Man SM . 2023. Guanylate‐binding proteins: mechanisms of pattern recognition and antimicrobial functions. Trends in Biochemical Sciences 48: 883–893. 37567806 10.1016/j.tibs.2023.07.002

[nph70524-bib-0016] Konopka CA , Schleede JB , Skop AR , Bednarek SY . 2006. Dynamin and cytokinesis. Traffic 7: 239–247. 16497219 10.1111/j.1600-0854.2006.00385.x PMC3654675

[nph70524-bib-0017] Mauxion JP , Chevalier C , Gonzalez N . 2021. Complex cellular and molecular events determining fruit size. Trends in Plant Science 26: 1023–1038. 34158228 10.1016/j.tplants.2021.05.008

[nph70524-bib-0018] Musseau C , Jorly J , Gadin S , Sørensen I , Deborde C , Bernillon S , Mauxion JP , Atienza I , Moing A , Lemaire‐Chamley M *et al*. 2020. The tomato Guanylate‐Binding Protein SlGBP1 enables fruit tissue differentiation by maintaining endopolyploid cells in a non‐proliferative state. Plant Cell 32: 3188–3205. 32753430 10.1105/tpc.20.00245 PMC7534463

[nph70524-bib-0019] Ngo CC , Man SM . 2017. Mechanisms and functions of guanylate‐binding proteins and related interferon‐inducible GTPases: roles in intracellular lysis of pathogens. Cellular Microbiology 19: e12791. 10.1111/cmi.12791 28975702

[nph70524-bib-0020] Ostler N , Britzen‐Laurent N , Liebl A , Naschberger E , Lochnit G , Ostler M , Forster F , Kunzelmann P , Ince S , Supper V *et al*. 2014. Gamma interferon‐induced Guanylate Binding Protein 1 is a novel Actin cytoskeleton remodeling factor. Molecular and Cellular Biology 34: 196–209. 24190970 10.1128/MCB.00664-13 PMC3911287

[nph70524-bib-0021] Pan L , Peng S , Zhang Y , Xu F , Cai X , Liang S , Huang Q , Yu S , Wang S . 2025. The nucleoporin CPR5 mediates plant immunity via guanylate‐binding proteins. Molecular Plant Pathology 26: e70086. 40288906 10.1111/mpp.70086 PMC12034427

[nph70524-bib-0022] Prakash B , Praefcke GJK , Renault L , Wittinghofer A , Herrmann C . 2000. Structure of human guanylate‐binding protein 1 representing a unique class of GTP‐binding proteins. Nature 403: 567–571. 10676968 10.1038/35000617

[nph70524-bib-0023] Ramachandran R , Schmid SL . 2018. The dynamin superfamily. Current Biology 28: R411–R416. 29689225 10.1016/j.cub.2017.12.013

[nph70524-bib-0024] Reimann TM , Müdsam C , Schachtler C , Ince S , Sticht H , Hermann C , Stürzl M , Kost B . 2023. The large GTPase AtGBPL3 links nuclear envelope formation and morphogenesis to transcriptional repression. Nature Plants 9: 766–784. 37095224 10.1038/s41477-023-01400-5

[nph70524-bib-0025] Rivas‐San Vicente M , Plasencia J . 2011. Salicylic acid beyond defence: its role in plant growth and development. Journal of Experimental Botany 62: 3321–3338. 21357767 10.1093/jxb/err031

[nph70524-bib-0026] Shydlovskyi S , Zienert AY , Ince S , Dovengerds C , Hohendahl A , Dargazanli JM , Blum A , Günther SD , Kladt N , Stürzl M *et al*. 2017. Nucleotide‐dependent farnesyl switch orchestrates polymerization and membrane binding of human guanylate‐binding protein 1. Proceedings of the National Academy of Sciences, USA 114: E5559–E5568. 10.1073/pnas.1620959114 PMC5514708 28645896

[nph70524-bib-0027] Sistemich L , Dimitrov Stanchev L , Kutsch M , Roux A , Günther Pomorski T , Herrmann C . 2021. Structural requirements for membrane binding of human guanylate‐binding protein 1. FEBS Journal 288: 4098–4114. 33405388 10.1111/febs.15703

[nph70524-bib-0028] Sistemich L , Kutsch M , Hämisch B , Zhang P , Shydlovskyi S , Britzen‐Laurent N , Stürzl M , Huber K , Herrmann C . 2020. The molecular mechanism of polymer formation of Farnesylated human guanylate‐binding protein 1. Journal of Molecular Biology 432: 2164–2185. 32087202 10.1016/j.jmb.2020.02.009

[nph70524-bib-0029] Solis‐Miranda J , Chodasiewicz M , Skirycz A , Fernie AR , Moschou PN , Bozhkov PV , Gutierrez‐Beltran E . 2023. Stress‐related biomolecular condensates in plants. Plant Cell 35: 3187–3204. 37162152 10.1093/plcell/koad127 PMC10473214

[nph70524-bib-0030] Spoel SH , Dong X . 2024. Salicylic acid in plant immunity and beyond. Plant Cell 36: 1451–1464. 38163634 10.1093/plcell/koad329 PMC11062473

[nph70524-bib-0031] Strambio‐De‐Castillo C , Niepel M , Rout MP . 2010. The nuclear pore complex: bridging nuclear transport and gene regulation. Nature Reviews Molecular Cell Biology 11: 491–501. 10.1038/nrm2928 20571586

[nph70524-bib-0032] Tang Y , Ho MI , Kang BH , Gu Y . 2022. GBPL3 localizes to the nuclear pore complex and functionally connects the nuclear basket with the nucleoskeleton in plants. PLoS Biology 20: e3001831. 36269771 10.1371/journal.pbio.3001831 PMC9629626

[nph70524-bib-0033] Tipton AR , Nyabuto GO , Trendel JA , Mazur TM , Wilson JP , Wadi S , Justinger JS , Moore GL , Nguyen PT , Vestal DJ . 2016. Guanylate‐Binding Protein‐1 protects ovarian cancer cell lines but not breast cancer cell lines from killing by paclitaxel. Biochemical and Biophysical Research Communications 478: 1617–1623. 27590579 10.1016/j.bbrc.2016.08.169 PMC5030188

[nph70524-bib-0034] Tourdot E , Mauxion JP , Gonzalez N , Chevalier C . 2023. Endoreduplication in plant organogenesis: a means to boost fruit growth. Journal of Experimental Botany 74: 6269–6284. 37343125 10.1093/jxb/erad235

[nph70524-bib-0035] Tretina K , Park E‐S , Maminska A , McMicking JD . 2019. Interferon‐induced guanylate‐binding proteins: Guardians of host defense in health and disease. Journal of Experimental Medicine 216: 482–500. 30755454 10.1084/jem.20182031 PMC6400534

[nph70524-bib-0036] Tripal P , Bauer M , Naschberger E , Mörtinger T , Hohenadl C , Cornali E , Thurau M , Stürzl M . 2007. Unique features of different members of the human guanylate‐binding protein family. Journal of Interferon and Cytokine Research 27: 44–52. 17266443 10.1089/jir.2007.0086

[nph70524-bib-0037] Uhlmann F . 2016. SMC complexes: from DNA to chromosomes. Nature Reviews Molecular Cell Biology 17: 399–412. 27075410 10.1038/nrm.2016.30

[nph70524-bib-0038] Unterer B , Wiesmann V , Gunasekaran M , Sticht H , Tenkerian C , Behrens J , Leone M , Engel FB , Britzen‐Laurent N , Naschberger E *et al*. 2018. IFN‐γ‐response mediator GBP‐1 represses human cell proliferation by inhibiting the Hippo signaling transcription factor TEAD. Biochemical Journal 475: 2955–2967. 30120107 10.1042/BCJ20180123 PMC6156764

[nph70524-bib-0039] Wandel MP , Pathe C , Werner EI , Ellison CJ , Boyle KB , von der Malsburg A , Rohde J , Randow F . 2017. GBPs inhibit motility of *Shigella flexneri* but are targeted for degradation by the bacterial ubiquitin ligase IpaH9.8. Cell Host & Microbe 22: 507–518. 29024643 10.1016/j.chom.2017.09.007 PMC5644667

[nph70524-bib-0040] Wang N , Wang Z , Tzourtzou S , Wang X , Bi X , Leimeister J , Xu L , Sakamoto T , Matsunaga S , Schaller A *et al*. 2022. The plant nuclear lamina disassembles to regulate genome folding in stress conditions. Nature Plants 9: 1081–1093. 10.1038/s41477-023-01457-2 PMC10356608 37400513

[nph70524-bib-0041] Wang S , Gu Y , Zebell SG , Anderson LK , Wang W , Mohan R , Dong X . 2014. A noncanonical role for the CKI‐RB‐E2F cell‐cycle signaling pathway in plant effector‐triggered immunity. Cell Host & Microbe 16: 787–794. 25455564 10.1016/j.chom.2014.10.005 PMC4282163

[nph70524-bib-0042] Wang W , Gu Y . 2022. The emerging role of biomolecular condensates in plant immunity. Plant Cell 34: 1568–1572. 34599333 10.1093/plcell/koab240 PMC9048959

[nph70524-bib-0043] Weismehl M , Chu X , Kutsch M , Lauterjung P , Herrmann C , Kudryashev M , Daumke O . 2024. Structural insights into the activation mechanism of antimicrobial GBP1. EMBO Journal 43: 615–636. 38267655 10.1038/s44318-023-00023-y PMC10897159

[nph70524-bib-0044] Wu Z , Guan KL . 2021. Hippo signaling in embryogenesis and development. Trends in Biochemical Sciences 46: 51–63. 32928629 10.1016/j.tibs.2020.08.008 PMC7749079

[nph70524-bib-0045] Zhang Y , Ge y , Sun K , Pan L , Liang Z , Wang P , Cai Y , Wan S . 2025. Identification and application of the heptad repeat domain in the CPR5 protein for enhancing plant immunity. Molecular Plant Pathology 26: e70059. 39910736 10.1111/mpp.70059 PMC11798864

[nph70524-bib-0046] Zhu S , Bradfield CJ , Maminska A , Park ES , Kim BH , Kumar P , Huang S , Kim M , Zhang Y , Bewersdorf J *et al*. 2024. Native architecture of a human GBP1 defense complex for cell‐autonomous immunity to infection. Science 383: eabm9903. 38422126 10.1126/science.abm9903 PMC12091997

